# Malignant tumors of the external auditory canal: diagnosis, treatment, genetic landscape, biomarkers, and clinical outcome

**DOI:** 10.37349/etat.2023.00169

**Published:** 2023-09-21

**Authors:** Pinelopi Samara, Michael Athanasopoulos, Anastasios Goulioumis, Ioannis Athanasopoulos

**Affiliations:** University of Toronto, Canada; ^1^Children’s Oncology Unit Marianna V. Vardinoyannis-ELPIDA, Aghia Sophia Children’s Hospital, 11527 Athens, Greece; ^2^Otolaryngology-Head and Neck Surgery, Karamandaneio Pediatric Hospital, 26331 Patras, Greece; ^3^Otolaryngology-Head and Neck Surgery, Athens Pediatric Center, 15125 Athens, Greece

**Keywords:** External auditory canal cancer, ear cancer, squamous cell carcinoma, ceruminous adenocarcinoma, surgery, radiotherapy, chemotherapy, biomarkers

## Abstract

Malignant tumors of the external auditory canal (EAC) are rare neoplasms that appear in the head and neck area. A common feature of these malignancies is their rarity, as well as their delayed diagnosis due to the appearance of non-specific symptoms that mimic various benign otologic conditions. The reported histological types of cancer of the external ear are: squamous cell carcinoma, basal cell carcinoma, malignant melanoma, Merkel cell carcinoma, angiosarcoma, adnexal carcinoma (including ceruminous adenocarcinoma and adenoid cystic carcinoma), and lymphoma (Lancet Oncol. 2005;6:411–20. doi: 10.1016/S1470-2045(05)70208-4). Several therapeutic interventions have been proposed, primarily orientated towards the cure of the patient, placing the surgical excision of the lesions at the tip of the spear. Subsequently and depending on the clinical stage and the pathological characteristics of the tumor, radiation, chemotherapy, a combination thereof, or some form of palliative treatment for particularly advanced cases, may be recommended. The aim of all the above-mentioned approaches is the complete resection of the mass with negative surgical margins along with lymph node dissection, the elimination of any residual disease or metastasis, and the improvement of survival. The anatomical complexity of the region will always remain a demanding challenge. Nevertheless, advances in the fields of ear microsurgery, imaging, radiation, molecular biology, and genomics have led to remarkable outcomes compared to the past, with a view to the patient’s quality of life. Large, well-organized, and prospective studies with the participation of multiple centers in contrast to existing retrospective studies with a limited number of patients will help to establish universally accepted guidelines. The exploration of the molecular and genetic background of these cancers in conjunction with the search for new biomarkers and target molecules seems promising for providing upgraded and more personalized treatment modalities for the future.

## Introduction

Ear tumors are traditionally divided into tumors of the outer ear, middle ear, and inner ear based on their location and are further classified into benign and malignant according to their proliferative/invasive potential. Cancers of the external auditory canal (EAC) are relatively rare, accounting for less than 0.2% of all head and neck cancers [1]. The EAC is the “bridge” between the auricle and the middle ear, extending from the lobe to the tympanic membrane. The size and shape of the canal differ among individuals, in adults it is approximately 2–3 cm long and 0.7 cm in diameter.

A number of important anatomical structures adjoin with the canal, namely the temporomandibular joint, the facial nerve, the internal carotid artery, and the internal jugular vein. EAC is lined by keratinizing squamous epithelium and cutaneous adnexal structures like hair follicles and sebaceous glands. Additionally, the outer portion of the external canal contains the ceruminous glands, a kind of modified apocrine glands that produce earwax. The pinna and the EAC are, in turn, supported by fibrofatty soft tissue, elastic cartilage, and bone [2].

Exostoses and osteomas are the dominant benign lesions of the EAC. It is known that serious damage could be caused even by benign tumors, as they are rather aggressive and locally destroy the sensitive region of the ear. The malignant lesions of the external ear are the following: squamous cell carcinoma (SCC), basal cell carcinoma (BCC), malignant melanoma, Merkel cell carcinoma (MCC), angiosarcoma (including both conventional angiosarcoma and Kaposi’s sarcoma) adnexal carcinoma (including ceruminous adenocarcinoma and adenoid cystic carcinoma) and lymphoma [3]. Malignant tumors of the EAC usually appear in people aged 40–60 years, or even older, and seem to affect males more than females. These tumors can extend from the EAC to the middle ear space, into the temporal bone, the skull base, or intracranially. The metastasis to EAC from other primary cancers is possible, but extremely uncommon. Depending on the stage of the disease and the treatment protocols, the 5-year survival rates range from 10–15% for advanced disease to 80–85% for early disease [4].

The main objective of this review is to present gathered data concerning a rare type of cancer, namely the EAC cancer, with an emphasis on subgroups, clinical manifestations, molecular basis, treatment, prognosis, biomarkers, and potential application of new molecular methods. The simple citation of previous studies with numbers and statistics, which are in the majority retrospective and concern a small group of patients, is beyond the scope of this manuscript.

## Management and guidelines for EAC cancers

The management of patients with this form of cancer remains challenging. The literature so far is limited due to the rarity of these carcinomas. Consequently, the published studies report small cohorts of patients with heterogeneous populations and no controlled clinical trials [4]. The recording of all information-regarding the clinical, histological, and imaging findings is necessary for the selection of the appropriate personalized treatment for each patient.

In order to plan the treatment protocol for a rare tumor, a reliable staging system is imperative. For cancer staging, the University of Pittsburgh revised Tumor-Node-Metastasis (TNM) system for EAC carcinoma is applied [1, 4]. The EAC tumor classification encompasses T1 for confined tumors, T2 for limited bone erosion or soft tissue involvement, T3 for osseous erosion with restricted soft tissue involvement or middle ear/mastoid extension, and T4 for extensive erosion or soft tissue involvement. Lymph node involvement is N0 for none and N1 for regional metastasis. Distant metastasis is categorized as M0 for none and M1 for its presence. Clinical stages are as follows: Stage I (T1N0M0), Stage II (T2N0M0), Stage III (T3N0M0 or T1N1M0), and Stage IV (T4N0M0, T2-4N1M0, or T1-4N0-1M1) [5]. Specifically, in the absence of a lymph node or distant metastasis, the tumor status determines the clinical stage. In the presence of lymph node metastasis, the disease is considered advanced and this finding indicates a poor prognosis. Even worse, distant metastasis denotes a very poor prognosis and is classified as stage IV disease.

The pathology report is essential, especially for rare neoplasms, and must be performed by experienced pathologists who will accurately analyze the specimens. Inexperienced pathologists should seek guidance or consult with experts regarding the biopsies arising from atypical or recurrent lesions of the EAC. Thus, the International Collaboration on Cancer Reporting (ICCR) has produced standardized and evidence-based data sets for various organs, including the ear and temporal bone. The data set provides guidelines for the reporting of the biopsy and the resection specimens, elements required for stage, prognosis, and treatment [6].

The 5th Edition of the World Health Organization Classification of Head and Neck Tumors: tumors of the ear discusses the tumors and tumor-like entities of the ear and temporal bone [7]. It correlates disease processes with clinical information and imaging, and highlights histological features and advances in the understanding of the molecular pathogenesis of these lesions [7].

## Diagnosis of EAC tumors

Some patients visit the otolaryngologist because of persistent symptoms, while others are diagnosed with a mass/lump/lesion during a routine ear exam. The steps that should be followed to establish the diagnosis are the following: otoscopy, audiometric testing-tympanometry, imaging, and biopsy-histological examination. A highly uncommon finding should be biopsied multiple times, if the first result is not indicative.

### Delay due to the special anatomy of the ear

EAC tumors are usually small and localized, making their diagnosis hard. Due to the complex anatomy of this area, not only it is often difficult to assess the extent of tumors clinically, but obtaining adequate biopsy material can be also tricky. Furthermore, since there is considerable overlap in clinical presentation, a thorough histopathologic evaluation of the tumor is necessary for the differential diagnosis. Ear cancers are often misdiagnosed, as they mimic other benign otologic conditions like otitis and cholesteatoma. Moreover, our experience has displayed that their low incidence, the lack of knowledge/experience from the physicians and the presence of non-specific symptoms are additional reasons that lead to misdiagnosis and consequently late intervention.

### Symptoms of patients with ear tumors

Typically, the symptoms depend on the size of the mass, the rate of growth, the degree of ear canal obstruction, and the potential infiltration into adjacent structures like vessels, nerves, and other solid constructions. The duration of symptoms ranges from a few months to several years. The most common symptoms are the following: fullness, hearing loss, pruritus, otalgia, otorrhea, bleeding, presence of mass, tinnitus, facial weakness, and facial palsy (Table 1). Patients may also present with symptoms consistent with chronic otitis media [1, 4]. As the abovementioned symptoms are non-specific, a detailed evaluation including a record of the patient’s medical history, clinical examination, full otoscopic/auditory evaluation, imaging techniques, and microbiological cultures of ear discharge is critical. We suggest that all patients with persistent otalgia/otorrhea or symptoms of chronic otitis that do not respond to conservative therapy are referred to an ear specialist for a thorough examination, regardless of their age.

**Table 1:** Symptoms of patients with EAC tumors

**Symptoms**
Fullness
Otalgia
Otorrhea/purulent ear discharge
Bleeding
Hearing loss
Pruritus
Tinnitus
Presence of mass/lump/ulceration
Facial weakness
Facial palsy

## Differential diagnosis and histologic types of malignant tumors of the EAC

Firstly, an EAC malignancy should be differentiated from the various benign tumors of the area including cystic chondromalacia and chondrodermatitis nodularis chronica helicis of the external ear, exostosis or osteoma, pleomorphic adenoma, syringocystoma papillferum and adenoma not otherwise specified (NOS) [7]. Moreover, vascular tumors and malformations of the EAC have also been defined, with otoscopy not being always sufficient for their characterization. Imaging should provide additional information about such masses, but the final and accurate diagnosis will be completed by the diagnostic biopsy and histology. Τhere are cases where the physical examination reported a benign lesion of EAC, but contrary to expectation, biopsy and histologic examination revealed the existence of carcinoma.

Given the rarity of cancer of the EAC, two are the “most frequently” occurring histologic types of EAC cancer: the SCC and the ceruminous gland adenocarcinoma; the main characteristics of which are described below.

### SCC

It is the most common type of cancer of the EAC (~80%). Histologically, the SCC of the external ear resembles that described in any other part of the human body. Specifically, the tumor cells present characteristic structures like infiltrating nests and cords or nests of polygonal cells with moderate amounts of eosinophilic cytoplasm and intercellular bridging. Suspicious lesions affecting the skin of the auricle are usually recognized on time. However, the covering of the ear canal is not easily visible, and access to examination and/or biopsy is limited. The surgery that aims at the auricle is directed to organ saving. Difficulties come in the identification of EAC lesions due to the inadequacy of biopsies and lack of expertise in the interpretation of the results. Surgery is the treatment of choice and in some advanced cases, extensive surgery will be required. Histological examination of the resection samples may be tricky due to the complex anatomy of the area. Similar practices could be employed for other tumors arising in the same site and requiring surgical involvement, besides SCC [7].

### Ceruminous gland adenocarcinoma

This group includes malignant tumors growing from the ceruminous glands of the external ear. Three distinctive histological subtypes are included: adenocarcinoma NOS, adenoid cystic carcinoma, and mucoepidermoid carcinoma. Adenoid cystic carcinoma, first described as “cylindroma”, accounts for almost 5% of EAC cancers. It has been described as confined to the middle ear and the mastoid. Histologically, adenoid cystic carcinomas might have a mixture of three distinctive growth patterns: cribriform, tubular, and solid [8]. To some extent, the predominant growth pattern is predictive of the clinical outcome. The tumors with principally tubular and cribriform morphology are low grade, whereas tumors with solid morphology are high grade. Of course, the patient’s prognosis is not only defined by the morphology. The treatment is surgical in this case as well. Fusions among specific genes, namely myeloblastosis (*MYB*), *MYB* proto-oncogene like 1 (*MYBL1*), and nuclear factor I B (*NFIB*), have been related to adenoid cystic carcinoma [9].

### Other even more rare malignant EAC tumors

BCC is another malignant tumor of the EAC (~5%). Histologically, BCC differs from SCC, as it is characterized by smaller cells, more hyperchromatic nuclei, mucinous stroma, and retraction artefact. MCC is a neuroendocrine malignant tumor which can be hardly found in the ear canal (~1%). Vesicular nuclei with small nucleoli, apoptosis, and abundant mitoses seem to be pathognomonic of MCC. Additional EAC malignancies include melanoma, lymphoma, and sarcoma (< 1%) [7].

## Improvement of the prognosis of EAC carcinomas

**Figure 1:**
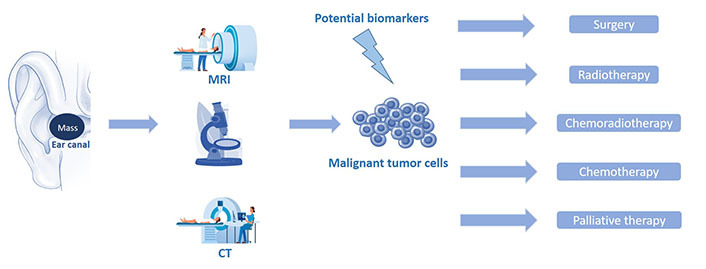
Schematic representation of the stages that mediate from the diagnosis of a malignant EAC tumor (imaging and pathology) to the patient’s combinatorial treatment. CT: computed tomography

The prognosis of such malignancies principally depends on the stage of the disease, as well as the administered therapy. Locally advanced tumors exhibit aggressive behavior and consequently less favorable outcomes. Surgery and adjuvant radiotherapy are the treatment of choice for the vast majority of these tumors providing improved survival for the patients. Early referral and aggressive primary surgical treatment with post-operative radiotherapy offer the greatest chance of cure. The most vital survival factor is the removal of the primary tumor with histologically clear margins. The development of microsurgery techniques and the introduction of innovative surgical equipment have also contributed to higher survival percentages compared to the past (Figure 1).

### Pre-operative radiological imaging’s role in EAC cancers

The new imaging systems, most frequently CT and magnetic resonance imaging (MRI), also play an important role in the diagnosis of ear tumors. More rarely, some patients are also tested using ultrasound (US), magnetic resonance angiography (MRA), and positron emission tomography (PET). In more detail, a very recent study by Nicoli et al. [10] in 87 patients with ear canal and middle-ear tumors revealed that pre-operative radiological imaging was used in the majority of patients with a malignant (90%) and a benign (80%) tumor. High-resolution CT and MRI with contrast agent was applied in 21% of benign and in 44% of malignant tumors. CT and MRI separately were used equally often for benign tumors, while CT and MRI were used in 28% *versus* 13% of malignant tumors. Regarding further imaging combinations, the ideal imaging choices were CT + MRI + MRA, CT + MRA, or US + CT + MRI in 12% of benign tumors and US  +  MRI or PET + CT + MRI in 6% of malignant tumors [10].

Generally, in clinical practice, the combination of CT + MRI appears as the ultimate imaging selection pre-operatively, as mentioned by Lee et al. [11] in a retrospective study of 56 patients with EAC carcinoma who underwent temporal bone resection and parotidectomy simultaneously. Indeed, high resolution CT and contrast-enhanced MRI are recommended for pre-operative planning. Using the scans, the ear, nose, and throat (ENT) surgeon can evaluate the extension of the tumor, as well as the penetration and erosion of the structures in order to design the surgical plan of excision. The surgeon will be fully organized in advance and will have the appropriate time to prepare the patient about what to expect after the operation. Of course, the exact extent of the damage and the potential infiltration of the adjacent area by the tumor will only be revealed during the surgery; as all these data could be either overestimated or underestimated in advance. The histological examination of the excised material and the close cooperation among surgeons, pathologists and oncologists will seal the final diagnosis. Certainly, similar imaging will also be programmed post-operatively in accordance with the following protocols for monitoring and early detection of any future recurrence.

### Assessing the role of surgery: the nature and extent of the operation

Surgical resection (en bloc or piecemeal) with tumor-free margin is the idyllic treatment, as positive surgical margins are synonymous with progressive disease. The therapeutic plan is defined by the clinical stage, the patient’s comorbidities, the patient’s wishes, and the doctor’s skills and preferences. For some stage T1 and all T2/T3 tumors, surgery (lateral temporal bone or subtotal temporal bone resection, both in combination with neck dissection and/or a parotidectomy) and adjuvant radiotherapy is the treatment of choice. For advanced T4 tumors, total temporal bone resection could be recommended, yet, it may cause serious complications without a definite increase in the survival rate. In a few cases, surgical treatment should be avoided; for example, in patients having severe comorbidities or extremely advanced tumors.

On the other hand, early-stage EAC cancers can be greatly cured by complete surgical excision and, hence, offer a long life to the patient [1]. Therefore, the post-therapeutic functional and cosmetic preservation is of high importance. The subsequent reconstruction of the canal with a tympanoplasty could help to maintain the best possible level of hearing, thus improving the quality of life [4]. Most studies, after the removal of the tumor following lateral temporal bone resection, proceed to reconstruction using skin grafts. Adequate size of the skin graft and blood supply to the graft bed is important for achieving a successful procedure [12]. As already mentioned, subtotal temporal resection could cause severe complications, such as hearing loss, elimination of the equilibrium function, or infections. Due to the delicacy of these surgical procedures, radiotherapy is often performed as an alternative therapy instead of surgery. Subtotal temporal bone resection is performed in patients with aggressive T3 or T4 tumors that could be removed en bloc and are without distant metastasis. Subtotal temporal bone resection is avoided in patients with severe comorbidities. For lymph node metastasis in the neck, patients without invasion to the carotid artery are indicated for surgery, even if the nodal status is in the advanced stage.

An interesting study particularly investigated the role of lateral temporal bone resection in the management of 36 patients (T1: 14, T2: 4, T3: 9, and T4: 9) with SCC of the EAC in terms of disease-free survival. For T1/T2 tumors, surgery succeeded with an excellent result. For T3 tumors, the surgery displayed good survival with a negative surgical margin when combined with pre-operative chemotherapy and/or post-operative radiotherapy. T3 patients who could not undergo the surgery had a poor prognosis, similar to that of T4 patients. So, besides subtotal temporal bone resection, lateral temporal bone resection could be a treatment option for some T3 patients [13].

### Determining the place of radiation in the treatment of EAC cancers

Radiotherapy is not feasible or indicated in every case. In general, it can be used in the following conditions: (a) patients who cannot go through surgery, (b) patients who underwent surgery but post-operatively present an increased risk of recurrence (for example existence of positive margins and locally advanced disease), and (c) patients bearing tumors that are operable and have locally infiltrated the area, as a pre-operative measure. The usual dose of post-operative radiotherapy is 50–60 Gy for T2–T4 tumors. Alternatively, some authors suggest 54–66 Gy for tumors with negative margins and 66–72 Gy for tumors with positive margins. For patients bearing inoperable tumors, 65–75 Gy is recommended as palliative therapy. In fact, the efficacy of CT-based three-dimensional conformal radiotherapy (3D-CRT) or intensity-modulated radiotherapy (IMRT) without surgery has been evaluated in 15 patients with SCC of the EAC, showing that chemoradiotherapy is inferior compared to surgery and post-operative radiation [14].

The “perfect” combination of approaches is still under examination, as there is disagreement among clinicians. However, it is generally accepted that especially in locally advanced cancers, local and systemic treatment should be integrated. Yet, there are patients who cannot receive surgery. So, to improve their prognosis, external-beam radiotherapy (EBRT) in conjunction with chemotherapy can be applied. Impressive results by Nagano et al. [15] from 21 patients with stage III–IV EAC cancer and Katano et al. [16] from 34 patients with stage I–IV EAC cancer suggest that the combination therapy of EBRT and surgery and/or chemotherapy may be the most effective treatment options for advanced EAC cancer. Actually, EBRT with simultaneous chemotherapy is the most acceptable pattern. Though, further investigation is needed to determine the optimal dose and the appropriate field for EBRT in these patients. Radiation-induced toxicities are usually late and concern bone or soft tissue necrosis around the ear, osteonecrosis of the jaw, and stenosis of the EAC.

### Has chemotherapy a role in the treatment of EAC cancer?

Several anticancer drugs, such as cisplatin, carboplatin, fluorouracil, docetaxel, and mitomycin, are widely administered. The docetaxel + cisplatin + 5FU (DCF) regimen seems the most effective method with high overall survival and reduced loco-regional recurrences. Some studies support the infusion of intra-arterial cisplatin in combination with radiotherapy for locally advanced cases [17]. Moreover, induction chemotherapy has been shown to convert potentially unresectable tumors to a resectable disease that could produce better outcomes.

## Radiation-induced EAC cancer: an existing entity

As already mentioned, radiation therapy is a vital part of the treatment of various head and neck tumors. The temporal bone and its related structures are frequently within the radiation field in the treatment of nasopharyngeal, oropharyngeal, parotid gland, and brain cancers. Thus, it has been found that radiation-associated malignancies (RAMs) have emerged. In order to name a malignancy as RAM, the following criteria must be met: the malignancies must arise in a prior-radiation field, they must present histologic characteristics discrete from those of the primary tumor, and occur at least 5 years after radiotherapy (malignancies 3–4 years after radiotherapy are also accepted as RAMs) [18]. The mutative effects of ionizing radiation have been identified for several years now: it causes breaks in the double-stranded DNA, leading to genomic instability. Achievement of negative surgical margins is especially important in patients with RAMs because re-irradiation can have harmful and often catastrophic effects, making radiotherapy in the post-operative setting extremely risky.

Given the rarity of cancers of the EAC and the even rarer occurrence of cancers in this area because of radiation, there are no well-established guidelines for the treatment of these malignancies. Resection to achieve negative margins when feasible and chemoradiation regimens, are just as important in RAMs as in non-RAMs [18]. Adjuvant re-irradiation as part of the treatment for RAM is a complex issue. To select or not to re-irradiate, certain features are evaluated among which are the state of the tissues, the amount of previously irradiated tissue removed, the reconstructive techniques, the prior-radiation dose, the time intervals between therapies, and the risk of recurrence. If post-operative re-irradiation is planned, microvascular free flap reconstruction is preferred. As cancer survival outcomes advance, clinicians should be aware of the potential long-term adverse effects of radiotherapy and include RAM in their differential diagnosis of patients with a history of radiation presenting with chronic symptoms in the ear and temporal bone.

## Other risk factors for the development of EAC cancer

For head and neck malignancies, including the EAC cancer, tobacco, and/or alcohol abuse act as risk factors. Moreover, chronic skin infections and chronic inflammation of the ear canal increase the risk of cancer [4]. Concerning SCC, constant exposure to the sun rises the risk of its occurrence. Additionally, several cases of SCC of the EAC secondary to cholesteatoma have been reported in the literature. Nevertheless, the role of cholesteatoma in the development of EAC carcinoma remains debatable, as some authors consider cholesteatoma as a risk factor, while others disagree [1].

## Molecular and genetic characteristics of EAC cancer

It is imperative to clarify the molecular characteristics of rare cancers, like EAC cancer, in order to understand the pathophysiology and the genetic behavior of the tumor, to translate all this information clinically and therefore establish innovative therapeutic proprieties. Recent studies for SCC of the EAC have recognized genetic drivers of rare malignancies, using whole exome sequencing (WES) and next‐generation sequencing (NGS) technologies, as well as bioinformatics tools. However, the genetic origin of this specific type of cancer and the mechanism that contributes to its appearance and progression have not been largely elucidated, in contrast to SCC in other primary sites.

Genetic alterations were recorded; the analysis of somatic mutations revealed that the most frequently altered gene is tumor protein p53 (*TP53*), followed by tyrosine kinase receptor genes and genes of phosphoinositide 3-kinase (*PI3K*) pathways, as well as amplifications of the chromosomal regions of 3q, 5p, and 8q [19]. Therefore, attempting a “primary” comparison of SCC, the most well-studied type of EAC cancer, with other cancers, as the evidence is limited, it is clear that *TP53*—the most often mutated tumor suppressor gene in human cancers is involved as well. In addition, the abovementioned genes have already been linked to the oncogenesis of many tumors, including head and neck cancer. Moreover, an independent group has reported an extremely rare case of synchronous bilateral primary SCC of the EAC highlighting the intra-patient heterogeneity. This incident is very special, as the tumor of the left ear was eliminated by the treatment, while the tumor of the right ear relapsed and progressed rapidly resulting in the death of the patient [20].

## Prognostic or predictive biomarkers for EAC cancer: are there any tangible data?

The term “biomarker” refers to DNA, RNA, proteins, metabolites, cytokines, or cell receptors that could potentially be used as a sign of a disease. The measurement of a biomarker in the biological fluids or tissues can determine the diagnosis of the disease, the monitoring of its course, the response to treatment, and the final outcome of the patient. The biomarkers that are currently being studied are numerous; yet, the biomarkers that are precise and clinically validated are very few. Although new biomarkers are constantly being developed in various diseases including head and neck cancer, as far as the ear is concerned, the data is minimal to zero [21]. The literature mentions biomarkers of the ear that relate to non-cancerous conditions, such as hearing loss and balance disorders [22]. Regarding cancer biomarkers, the majority of the studies focus on other types of head and neck carcinomas like oropharyngeal or nasopharyngeal. Recently, studies have focused on SCC of the EAC, but unfortunately, no specific biomarker has entered the clinical practice so far. Therefore, the onset and development of SCC should be further investigated. Data from screening and analysis of long non-coding RNAs (lncRNAs) in patients with SCC of the EAC shed light on the pathogenesis of this rare disease. Specifically, lnc-MMP3-1 seems to be a novel survival predictor for these patients [23].

## Limitations of the existing literature concerning EAC cancer

Unfortunately, existing studies are subject to various limitations, most of which stem from the rarity that characterizes these types of cancer. The most important limitation is the low number of reported cases, as well as the retrospective nature of the studies which could have produced a selection bias. Moreover, the wide heterogeneity of the treatment modality and the different pathological types may have resulted in inaccurate results.

## Conclusions

Diagnosis and treatment of cancers of the EAC is particularly challenging, as the complex anatomy of the area does not favor their timely detection. Clinicians and principally otolaryngologists should be particularly suspicious of chronic or repeated lesions, as well as lesions following former irradiation of the surrounding area. Early discovery of the mass and multimodal treatment will lead to the best possible outcome for the patient. Focusing on the identification of potential mutations involved in ear/ear canal carcinogenesis and the development of new biomarkers will offer sooner or later new targeted cancer therapies.

## Abbreviations

BCC: basal cell carcinoma
CT: computed tomography
EAC: external auditory canal
EBRT: external-beam radiotherapy
MRA: magnetic resonance angiography
MRI: magnetic resonance imaging
MCC: Merkel cell carcinoma
RAMs: radiation-associated malignancies
SCC: squamous cell carcinoma
US: ultrasound

## Declarations

### Copyright

© The Author(s) 2023.
